# Evaluation of the European Foundation Initiative into African Research in Neglected Tropical Diseases by the African Fellows

**DOI:** 10.1371/journal.pntd.0002019

**Published:** 2013-03-14

**Authors:** Hester G. O'Neill, Themba Mzilahowa, Nilsa de Deus, Sammy M. Njenga, Elia J. Mmbaga, Thomas M. Kariuki

**Affiliations:** 1 Department of Microbial Biochemical and Food Biotechnology, University of the Free State, Bloemfontein, South Africa; 2 Malaria Alert Centre, Malawi College of Medicine, Blantyre, Malawi; 3 Instituto Nacional de Saúde, Maputo, Mozambique; 4 Eastern and Southern Africa Centre of International Parasite Control, Kenya Medical Research Institute, Nairobi, Kenya; 5 Department of Epidemiology and Biostatistics, Muhimbili University of Health and Allied Sciences, Dar es Salaam, Tanzania; 6 Institute of Primate Research, National Museums of Kenya, Nairobi, Kenya; Ministère de la Santé Publique et de la Lutte contre les Endémies, Niger

## Neglected Tropical Diseases in Africa

Neglected tropical diseases (NTDs) continue to adversely affect the lives of millions of people across the African continent. A roadmap published by the World Health Organization (WHO) in early 2012 lays out a vision for the control, elimination, and eradication of 17 NTDs [1]. One of the goals of this roadmap is to encourage the community of partners, including donors, pharmaceutical companies, agencies, nongovernmental organizations (NGOs), philanthropists, and universities, to maintain and expand their commitments to overcome the scourge of NTDs. Of the 17 WHO-listed NTDs, 14 are found in Africa and some are exclusively African diseases, e.g., human African trypanosomiasis. It is widely argued that priority setting, research and development of tools and solutions, as well as operational and implementation strategies for NTDs should involve African scientists and communities.

## Funding Research in NTDs

Over the last decade or two, most of the global funding mechanisms have been directed at HIV/AIDS, malaria, and tuberculosis [2]. However, at least 1 billion people are affected by NTDs globally, with less than 1% of health-development support allocated for NTDs [3], [4]. This trend is changing, thanks largely to the efforts of donors and foundations. Following the recent London Declaration to Combat NTDs (www.unitingtocombatntds.org), which includes commitments for support from governments, foundations, and pharmaceutical companies, it is expected that much-needed impetus to address some of the current challenges will be provided. In addition, it has been suggested that the traditional approach to undertaking and supporting research in Africa requires a change of mind-set from both the scientists and funding organizations. This change of mind-set from the traditional approach should include a more integrated approach regarding funding organizations and the formation of sponsor-scientist partnerships [5].

## European Foundation Initiative into African Research in Neglected Tropical Diseases (EFINTD)

In 2008, five European foundations (the Volkswagen, Gulbenkian, Mérieux, Nuffield, and Cariplo foundations) driven by a common goal formed the EFINTD (www.ntd-africa.net). EFINTD aims to strengthen research capacity in NTDs in Africa. This objective is accomplished by harnessing the skills of and empowering young African researchers who are based in their respective countries to perform research on various NTDs. The funding scheme supports recently qualified doctorate researchers with junior fellowships (90,000 EUR) and more advanced researchers with senior fellowships (140,000 EUR). Selection of fellows is based on peer review of a full grant application, followed by defence of the proposals at a grantees meeting convened by EFINTD. At the grantees meeting, part of the selection process is an interview conducted by eminent scientists in the field of NTDs and donor representatives. The duration of the fellowships, which also include a mentorship programme, is three years. The successful fellow would continue with normal duties such as clinical or teaching responsibilities for the duration of the fellowship. African host institutions must provide the basic infrastructure and support for the project, although no commitment is required for career support beyond the life of the fellowship. Career growth of the African scientists is supported by offering financial support for direct project expenses, such as personnel, equipment, consumables, as well as facilitating yearly meetings, workshops, discussion groups, networks, and local and regional African-led collaborations. A contribution to administrative costs payable to the host institution is limited to a maximum of 3,000 EUR.

To date three rounds (2008, 2010, and 2012) of fellowship awards (24 in total) have been dispensed. Twelve awards involve research into tool-ready diseases such as lymphatic filariasis, onchocerciasis, schistosomiasis, and soil-transmitted helminthiases. Seven awards focus on tool-deficient diseases such as Buruli ulcer and human African trypanosomiasis [6]. Four awards are for virus research, focusing on rotavirus, Lassa virus, and arbovirus infections. One fellowship was awarded for bacterial meningitis. In addition, three junior fellows awarded in 2008 received senior fellowship awards in 2012.

## Evaluation of the EFINTD Fellowship Programme

After three years, it is useful to ask whether the approach taken by EFINTD is feasible, effective, and efficient. It is common practice in many donor-supported programmes and initiatives for fellows to be silent recipients and beneficiaries rather than drivers of the programme direction. Programme reviews or monitoring and evaluation exercises are usually done by external advisers and/or consultants. Although this is standard practice, it does not reflect on the experiences of the fellows. Therefore, using SurveyMonkey (www.surveymonkey.com), an online survey and questionnaire tool, a survey was conducted among the recipients of the first two rounds (2008 and 2010) of the EFINTD fellowships (Table S1). The survey was developed using preconfigured questions from the SurveyMonkey question library. These included multiple choice, matrix choice, and rating-scale questions, as well as text boxes that allowed fellows to provide additional information (Survey S1). The survey was opened on September 23, 2011, and a survey link was forwarded to the fellows by SurveyMonkey. Two reminders were sent on October 17 and again on November 15. The survey was finally closed and the data collected on November 22, 2011. All 19 fellows that received the link completed the survey, representing 10 African countries. Additional information regarding the mentorship programme, peer-reviewed publications by the fellows, and conference attendances was sourced via e-mail and at a grantees meeting. Information regarding the influence of fellows' projects on local policies was also collected by e-mail. The results of the survey are summarised here and aim to provide the first evaluation of the EFINTD programme by the African fellows.

### 1. Demographic Information

At the time of the survey, 19 fellowships had been awarded (Table S1). These comprised nine senior fellows and ten junior fellows. West and East Africa are the best represented regions with seven fellows each, while three fellows are based in Southern Africa and two fellows in Central Africa. Four of the fellows are female, highlighting the low number of postdoctoral female scientists on the continent. Two of the female fellows are based in Southern Africa and two in East Africa. Four fellows are from Ghana, three from Kenya, and three from Uganda. Anglophone countries are the most successful (15 fellows), while three fellows originate from Francophone countries and one fellow from a Portuguese-speaking country, emphasizing the challenges faced by non-Anglophone scientists in overcoming language barriers and accessing English-based donor programmes.

### 2. Impact of the EFINTD Fellowship Programme

#### 2.1 Outputs

**Figure 1:**
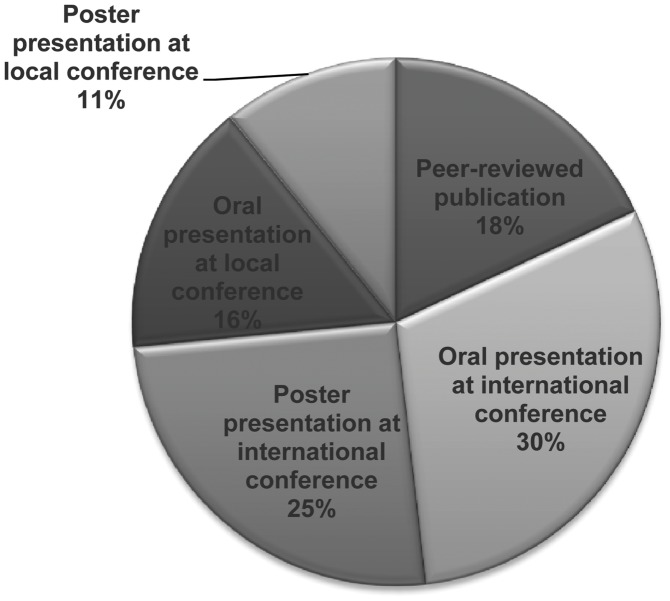
Scientific dissemination of research findings by the 2009 and 2010 EFINTD fellows.

Since the first awards were made in 2008, several outputs have been generated by the fellows, as summarised in Figure 1. Fifteen peer-reviewed papers [7]–[21] have been published with an average impact factor of 2.87 (range 1.38–4.72) (Table S2). In total the 15 papers have received 27 citations since the first papers were published in 2010. The paper by Ekpo and co-workers [8], published in *Parasites and Vectors* in 2010, received the highest number of citations with 7 citations. Almost half (47%) of these publications have been in open-source journals, reflecting the shared philosophy of the fellows to communicate their research results to the widest possible audience, which is especially relevant for African researchers and institutions with limited resources and, subsequently, paid journal subscriptions. More than half of the total outputs generated by the fellows were contributions at international conferences, either as oral or poster presentations, which is in-line with the desire of the fellows and sponsors to disseminate research outputs as quickly as they are generated.

#### 2.2 Scientific capacity building and career development

Although 19 fellowships were awarded, more than 60 additional students and personnel were employed on the various EFINTD-funded projects. These include 31 students (at MSc and PhD level) as well as 31 staff members who ranged from medical and veterinary doctors to laboratory technicians and human resource personnel. Eight fellows indicated that the fellowship programme impacted positively on their careers, either in the form of promotion or appointment to new positions.

The EFINTD funding also allowed for fellows to attend and present at project-topic-related workshops and courses at leading European institutions such as the Bernhard Nocht Institute for Tropical Medicine in Hamburg, the Institute of Tropical Medicine in Antwerp, the Swiss Tropical and Public Health Institute in Basel, the Karolinska Institute in Stockholm, the University of Copenhagen in Copenhagen, and the Royal Tropical Institute in Amsterdam. Visits to the United States (Harvard University and Louisiana State University), the Middle East (King Saud University, Riyadh), and other African countries were also undertaken. It was also possible to visit collaborators and other laboratories to learn/master new techniques. In addition, one fellow attended the 60^th^ Lindau Nobel Laureate Meeting in 2010, while three fellows attended the 61^st^ Lindau Nobel Laureate Meeting in 2011. Seven of the fellows were also able to secure additional funds by using the EFINTD funding as leverage.

Another important aspect of the programme has been the commitment of EFINTD to so-called “soft-skills” development. A weeklong summer school at the Royal Tropical Institute in Amsterdam attended by most of the fellows is testimonial of this commitment. The summer school included topics such as leadership, project management, financing NTD research, ethics and human subject research, epidemiology, evidence-based healthcare, as well as reporting and publishing results. In addition, several workshops are routinely held at the grantees meetings, dealing with issues such as prioritizing research activities for NTDs and networking research activities. The impact of such programmes goes beyond empowering the fellows, as they have indicated that the skills and knowledge they acquire are quickly transferred to their research groups, laboratories, institutes, and university departments.

#### 2.3 Infrastructure development

A question regarding the contribution of the programme to infrastructure (equipment and laboratory space) had an overwhelmingly positive response, with more than 75% of the fellows indicating that they had benefited in this respect. Although, in most instances, the contribution involved only small equipment (thermal cyclers, microcentrifuges, etc.) and computers (hardware and software), these resulted in a significant impact at the respective institutions by enhancing the capacity of the research groups. In two instances, additional grants were secured with the help of EFINTD that enabled the fellows to upgrade their facilities to regional reference laboratories based in Kenya and Malawi.

#### 2.4 Impact on policy

Although the projects have not been running for an extensive period, influence on local policies is already evident. At a local government level, this ranges from assistance to local districts (e.g., the decentralized control of animal reservoirs for trypanosomiasis in Uganda) to awareness and educational programmes in Nigeria for public health officials on the primary transmission of Lassa virus from the multimammate rat to humans. At a national level, scientists are also participating in training. Specifically this has been the case in Kenya where a study coordinator also trains programme implementers for the national Schistosomiasis Control Programme. The provision of maps describing the spatial distribution of schistosomiasis in Nigeria to national governmental departments and NGOs has impacted on policy and the national schistosomiasis control programme in this country. In addition, it has helped to accelerate and delineate areas for drug distribution and treatment. Geospatial and mapping support generated by fellows have also been provided to the NTD Control Programme in Nigeria.

#### 2.5 Networking

The EFINTD programme has brought together scientists from different scientific backgrounds with various skills and expertise to forge local collaborations. Subsequently, scientific capacity has been generated and is being harnessed in combating specific NTDs in African countries, where a dire need still exists. The importance of South–South networking in the improvement of scientific capacity in Africa was highlighted in a recent report by the Disease Reference Group on Helminth Infections [22]. Therefore, the development of such an extensive South–South network enriches the African research landscape and will hopefully have a long-term impact and be a valuable resource. In addition, North–South networking is encouraged through the mentorship programme and grantees meetings, which allow for formal and informal interaction with renowned scientific experts and sponsors.

### 3. Mentorship Programme

A mentorship support programme is offered to the successful fellowship applicants. The mentorship programme allows fellows to select and establish links with leading African and European scientists, thereby enabling fellows to select their own mentor(s). Resources (10,000 EUR) are provided for the fellows to visit their mentors and vice versa. Several of the fellows responded positively with regard to their experiences in the mentorship programme, and one fellow stated “I have benefitted from it immensely.” This was especially the case in terms of advice and support received in managing current projects and providing ideas for future directions thereof as well as career advancement and growth towards becoming independent scientists. The programme was rated as enriching and unique, since it differs from the traditional model of mentoring in which a mentor is assigned to a fellow. This model has been widely adopted by other agencies and African universities, but with less positive results. What also makes the EFINTD mentorship programme special is that it has a built-in mechanism to follow up on both the mentor and mentee. In the end both parties are accountable firstly to themselves, to each other, and lastly to the foundations. The fellows were, however, quick to emphasize that the success of this relationship is dependent on the choice of mentor, his/her availability, and personality.

### 4. Overall Rating of the EFINTD Fellowship Programme

**Figure 2:**
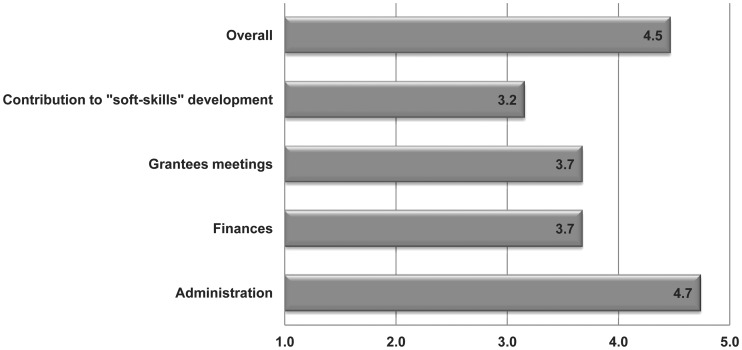
Average rating of the EFINTD fellowship programme. Rating was provided in terms of administration, finances, grantees meetings, “soft-skills” development, as well an overall rating of the programme. The average rating scores achieved for each parameter are indicated. Rating scale: 1-very poor; 2-poor; 3-neutral; 4-good; and 5-very good.

The overall rating of the fellowship programme by the fellows was overwhelmingly positive (Figure 2). Their appreciation for the programme is emphasized by the following quotes from the fellows:

- “It is the personal-development aspect of the EFINTD postdoctoral programme that I like best.”
- “The fellowship is exceptional as it provide fellows with opportunities to study in-country while obtaining necessary technical assistance from anywhere in the world.”
- “…in terms of flexibility and access to funds as well as support, this grant is excellent.”

The ratings indicated that in terms of flexibility in administration of and access to funds, this programme has been outstanding. The programme is seen as unique, as it provides opportunities for African researchers to perform research on NTDs in their respective home countries and institutions. Furthermore, it is now possible for African researchers to apply for and access European funds directly and not through a European partner. A revision of the budget limits was, however, suggested to accommodate student and infrastructure support as well as foreign currency fluctuations. More emphasis on “soft-skills” development, especially in terms of career development, should also be encouraged.

## Conclusions and Recommendations

The consensus among the 19 fellows is that the EFINTD fellowship programme is a prestigious postdoctoral programme that is highly competitive and has a transformational research agenda. Furthermore, the EFINTD programme has empowered the fellows and their home institutions to effectively undertake and contribute to research on NTDs, raised their individual profiles in the NTD arena, and created opportunities to establish long-lasting local and international partnerships. The success of the EFINTD fellowship programme in Africa can be attributed to the following aspects:

- Empowerment of African scientists to perform research in-country on African problems
- Direct access to research funds
- An effective mentorship programme that allows for fellows to select their own mentor
- Access to and creation of networking opportunities
- Investment in “soft-skills” development by funders
- Close contact between funders and fellows, facilitated by regular grantees meetings and workshops

In turn the African scientists feel that the foundations have benefited from the in-depth perspectives brought by researchers who come from diverse cultures, backgrounds, and NTD challenges through their very close personal contacts with the fellows. Therefore, they have been able to ensure that their investments are paying dividends and have a better appreciation of the magnitude of these diseases in an African context. Since this is a dynamic and developing programme, the fellows made several suggestions and recommendations for its future. These suggestions might also be of relevance to other funding agencies with tailor-made programmes for African researchers:

- Minimum duration of programme. The fellows recommended continuation and possible extension of the current programme. Currently both junior and senior fellowships have a three-year duration. However, recruitment and delivery of PhD students can be problematic within three years. Although it is possible for senior fellows nearing the end of their tenure to apply for second-phase funding, which provides an additional two years of funding, it would be more desirable to extend the duration of the senior fellowship from three to five years, with intermediate progress review after three years.
- Long-term support for fellows that excel during the initial two rounds of funding. This could involve either the implementation of a third funding phase (career development into research leader) or facilitating support from other funding agencies, such as the Bill and Melinda Gates Foundation, the Wellcome Trust, or the European Commission.
- Sustainable long-term South–South network development. Such initiatives are already being developed and include an EFINTD networking group within the GLOBE e-portal of Foundation Mérieux. Another initiative that is currently being explored is the formation of an academy or association of African NTD researchers that would allow recipients and donors to continue interacting beyond the life of individual projects.
- Capacity building in terms of postgraduate students (MSc and PhD). Student bursaries can be incorporated into the current fellowships to allow student training and provide access to some of the benefits of the fellowship programme. In addition, it is recommended to transfer the current postdoctoral programme to MSc and PhD level. Furthermore, it is suggested to promote such a programme, especially in non-English speaking countries and among women scientists, in order to increase the scientific capacity in Africa.
- Infrastructure development in terms of equipment grants. A major consideration should be to develop programmes that specifically target infrastructure upgrades in African institutions, and these should be separate from research project support. Such upgrades would allow for independent research and the improvement of the research environment, which should enable African researchers to become internationally more competitive and attract back to Africa the large pool of highly skilled African scientists residing in the diaspora.
- Continuation and expansion of “soft-skills” development to include topics such as career planning and development.
- Establish communication channels with other national stakeholders or decision makers in government departments through workshops, etc.
- Publication of outcomes and main research findings generated during the EFINTD programme. This could be in the form of annual publication reports, information bulletins, or establishment of a formal peer-reviewed journal under the EFINTD Initiative.

## Supporting Information

Survey S1 **SurveyMonkey questionnaire.**
(DOCX)

Table S1 **Description of 2008 and 2010 EFINTD fellows that participated in the survey.**
(DOCX)

Table S2 **Description of peer-reviewed papers related to EFINTD projects published by 2008 and 2010 fellows.**
(DOCX)
