# Supplementary material for: Evaluation of the European Foundation Initiative into African Research in Neglected Tropical Diseases by the African Fellows
Source: PLoS Negl Trop Dis. 2013 Mar 14;7(3):e2019. doi: 10.1371/journal.pntd.0002019 (PMC3597492; doi:10.1371/journal.pntd.0002019)
Supplement: Survey S1 — SurveyMonkey questionnaire. (DOCX) [file pntd.0002019.s001.docx]

**Survey S1 – SurveyMonkey Questionnaire**

*Indicate compulsory questions.
