# Supplementary material for: Evaluation of the European Foundation Initiative into African Research in Neglected Tropical Diseases by the African Fellows
Source: PLoS Negl Trop Dis. 2013 Mar 14;7(3):e2019. doi: 10.1371/journal.pntd.0002019 (PMC3597492; doi:10.1371/journal.pntd.0002019)
Supplement: Table S1 — Description of 2008 and 2010 EFINTD fellows that participated in the survey. (DOCX) [file pntd.0002019.s002.docx]

**Table S1: Description of 2008 and 2010 EFINTD fellows that participated in the survey.**

| Name | Fellowship Awarded | Year | Country | Project title |
| --- | --- | --- | --- | --- |
| A. Ablordey | Senior | 2010 | Ghana | Scaling up early detection and treatment to reduce Buruli ulcer morbidity in the Asante Akim North District of Ghana |
| A.Y. Debrah | Junior | 2008 | Ghana | Analysis of genetic polymorphisms of cytokines, vascular endothelial growth factors (VEGFs) and VEGF receptors in disease manifestations in lymphatic filariasis |
| N. De Deus | Junior | 2010 | Mozambique | Comparative study of rotavirus infection between HIV positive and negative children less than 5 years with acute and severe diarrhea |
| U.F. Ekpo | Junior | 2008 | Nigeria | Development of a nationwide geographical information system database and Bayesian spatial predictive models for the control of Schistosomiasis in Nigeria |
| T.M. Kariuki | Senior | 2008 | Kenya | Analysis of protective immune responses to Schistosomiasis in a primate model |
| M. Koffi | Junior | 2010 | Ivory Coast | Investigation of the role of trypanolytic factor (TLF) genes in the control of trypanaosome infection in humans |
| *E. Matovu | Senior | 2008 | Uganda | Genetic characterization of trypanosomes isolated from humans, tsetse flies and animal reservoirs in the Trypanosoma brucei gambiense-T. b. rhodesiense interface districts of Northern Uganda |
| E.J. Mmbaga | Junior | 2010 | Tanzania | Female genital schistosomiasis and HIV-1 infection in Lower Moshi, Kilimanjaro, Tanzania |
| P. Mwinzi | Senior | 2010 | Kenya | Implementation of community directed intervention (CDI) for schistosomiasis and soil-transmitted helminthiasis (STH) in an urban setting: Western Kenya |
| T. Mzilahowa | Senior | 2010 | Malawi | Vector competence and filariasis transmission in Malawi |
| *A.J. Nalunkuma  Kazibwe | Junior | 2008 | Uganda | Genetic characterization of trypanosomes isolated from humans, tsetse flies and animal reservoirs in the Trypanosoma brucei gambiense-T. b. rhodesiense interface districts of Northern Uganda |
| S.M. Njenga | Senior | 2008 | Kenya | Integration of mass drug administration strategies for three neglected tropical diseases in Malindi District, Kenya: Schistosomiasis, soil-transmitted helminthiasis and lymphatic filariasis |
| A. Olayemi | Junior | 2010 | Nigeria | Ecology of transmission of the deadly Lassa virus from rodents to man across various flashpoints within Nigeria |
| H.G. O’Neill | Senior | 2008 | South Africa | Generating regional rotavirus vaccine strains through reverse genetics to alleviate viral diarrhea in Africa |
| M. Osei-Atweneboana | Senior | 2010 | Ghana | Development of genetic markers for early detection and monitoring of Ivermectin resistance in Onchocerca volvulus and its implication for onchocerciasis control |
| R.O. Phillips | Senior | 2008 | Ghana | Development of an oral regimen for treatment of Buruli ulcer |
| G. Simo | Junior | 2008 | Cameroon | Study of the population genetic structure of Glossina palpalis palpalis: Implications in the improvement of the control strategies of sleeping sickness in central Africa |
| N. Tendongfor | Junior | 2008 | Cameroon | Use of BALB/c mice genetically deficient in cytokine receptor genes to study the immunological mechanisms involved in the survival and development of Loa loa. O. volvulus and M. perstans filariae in the vertebrate host |
| R. Tweyongyere | Junior | 2008 | Uganda | Does praziquantel treatment of schistosomiasis during pregnancy influence immune responses to S. mansoni infection among children born to the treated women? |

*A senior/junior partnership was awarded to Drs. Matuvo and Kazibwe.
