# Supplementary material for: Evaluation of the European Foundation Initiative into African Research in Neglected Tropical Diseases by the African Fellows
Source: PLoS Negl Trop Dis. 2013 Mar 14;7(3):e2019. doi: 10.1371/journal.pntd.0002019 (PMC3597492; doi:10.1371/journal.pntd.0002019)
Supplement: Table S2 — Description of peer-reviewed papers related to EFINTD projects published by 2008 and 2010 fellows. (DOCX) [file pntd.0002019.s003.docx]

Table S2: Description of peer-reviewed papers related to EFINTD projects published by 2008 and 2010 fellows.

| Reference | Journal Title | Impact Factor* | Open Access | Year of publication | Citations^#^ |
| --- | --- | --- | --- | --- | --- |
| Debrah et al [7] | Human Immunology | 2.84 | No | 2011 | 1 |
| Ekpo et al [8] | Parasites and Vectors | 2.94 | Yes | 2010 | 7 |
| Ekpo et al [9] | Journal of Helminthology | 1.38 | No | 2012 | 0 |
| Ekpo et al [10] | Parasitology | 2.96 | No | 2012 | 0 |
| Farikoua et al [11] | Acta Tropica | 2.72 | No | 2010 | 4 |
| Hürlimann et al [12] | PLoS Neglected Tropical Diseases | 4.72 | Yes | 2011 | 3 |
| Melachio et al [13] | Parasites and Vectors | 2.94 | Yes | 2011 | 1 |
| Mlera et al [14] | Journal of Virological Methods | 2.01 | No | 2011 | 3 |
| Njenga et al [15] | Parasites and Vectors | 2.94 | Yes | 2011 | 0 |
| Njitchouang et al [16] | Medical and Veterinary Entomology | 1.91 | No | 2011 | 1 |
| Odiere et al [17] | Parasitology | 2.96 | No | 2011 | 3 |
| Opisa et al [18] | Parasites and Vectors | 2.94 | Yes | 2011 | 1 |
| Sarfo et al [19] | PLoS Neglected Tropical Diseases | 4.72 | Yes | 2011 | 1 |
| Simo et al [20] | Experimental Parasitology | 2.12 | No | 2011 | 2 |
| Tendongfor et al [21] | Parasites and Vectors | 2.94 | Yes | 2012 | 0 |

*Impact Factors are indicated according to Thomson Reuters Web of Knowledge Journal Citations Reports® for 2011

^#^Citations are presented as indicated in Scopus®
